# The effect of ethylene glycol on pore arrangement of anodic aluminium oxide prepared by hard anodization

**DOI:** 10.1098/rsos.171412

**Published:** 2018-03-07

**Authors:** Yang Guo, Li Zhang, Mangui Han, Xin Wang, Jianliang Xie, Longjiang Deng

**Affiliations:** 1 National Engineering Researching Center of Electromagnetic Radiation Control Materials, University of Electronic Science and Technology of China, Chengdu 610054, People's Republic of China; 2 State Key Laboratory of Electronic Thin Films and Integrated Devices, University of Electronic Science and Technology of China, Chengdu 610054, People's Republic of China; 3 Key Laboratory of Multi-spectral Absorbing Materials and Structures of Ministry of Education, University of Electronic Science and Technology of China, Chengdu 610054, People's Republic of China

**Keywords:** anodic aluminium oxide, hard anodization, ethylene glycol, pore arrangement

## Abstract

The influence of the addition of ethylene glycol (EG) on the pore self-ordering process in anodic aluminium oxide (AAO) membranes prepared by hard anodization (HA) was investigated. It was illustrated that EG has a substantial effect on the pore arrangement of AAO, and it was found that a smaller pore size can be obtained with an EG concentration reaching 20 wt% in aqueous electrolyte. The number of estimated defects of AAO increases significantly with an increase in EG concentration to 50 wt%. Excellent ordering of pores was realized when the samples were anodized in the 30 wt%-EG-containing aqueous electrolyte.

## 1. Introduction

Anodic aluminium oxide (AAO) has drawn widespread attention due to its well-defined pore architecture and suitable corrosion resistance, thermal stability, hardness, abrasion resistance and insulation properties [1–4]. Meanwhile, as a nano-template or host material, AAO plays an essential role for various surface engineering applications, e.g. molecular separation, catalysis, energy storage, electronics, sensors, drug delivery and template synthesis [5,6], and is a component in a diverse range of nanostructured materials in the form of nanodots, nanowires and nanotubes [7,8].

AAO can be fabricated by hard anodization (HA) in various aqueous electrolytes [8]. The HA process has an advantage of high-speed growth ascribed to the high current density (*j*) with increased anodizing potential (*U*). Nevertheless, however, AAO made by the HA process demonstrated severely buckled surfaces with numerous cracks and distorted pores [9–11]. Moreover, the controllability of pore diameter (*D*_p_) and the aspect ratio of nanopores of AAO was also very low.

To solve the problems in the process of HA, organic compounds were added to the aqueous electrolyte, which aimed at prompting the ordered arrangement of AAO [12–15]. Zaraska *et al.* [12] have reported the results of fabrication of AAO with electrolyte containing different *n*-alcohols, and showed that alcohol can boost parameter regularity and pore circularity. Besides, Chen *et al.* [13] used polyethylene glycol (PEG) as a modulator to manipulate pore sizes of AAO. The phenomenon of the presence of PEG in the electrolyte restrained chemical dissolution of alumina (Al) and resulted in smaller pores. Meanwhile, AAO could be fabricated by a non-lithographic approach as reported by Martin *et al.* [14], which illustrated that EG had the ability to improve parameter regularity and lower the pore diameter of AAO. Recently, the addition of organic compounds in the formation of AAO has received much attention. Vega *et al.* [4] commented on the preparation of AAO by HA in a renewed electrolyte with different oxalic acid and ethanol concentrations and explained the role of oxalate ions and their limited diffusion through alumina nano-channels from a bulk electrolyte on the basis of a phenomenological model. Furthermore, Norek *et al.* [15] have also reported a systematic study on the effect of addition of EG to oxalic acid electrolytes for the HA process of Al.

In this paper, the effect of EG concentration on the pore arrangement of AAO produced by HA in oxalic acids is analysed. The results demonstrate that the presence of EG has a significant effect on pore ordering and pore size during the HA process. Simultaneously, the results shed new light on the pore-ordering phenomena during HA.

## 2. Experiment

Electro-polished ultrapure aluminium foils (greater than 99.999% Al) were cut into square samples of size 3.3 cm × 3.3 cm. The Al samples were embedded in an insulated loader and served as the anode. The back and edges of the loader were sealed with plastic. Graphite was used as the cathode and the distance between both electrodes was kept constant (approx. 4.5 cm). A 0.3 M oxalic acid solution was used as the electrolyte for all samples. A calculated amount of EG was also added to the solutions with the final EG concentrations being 0, 10, 20, 30, 40 and 50 wt% and labelled as *S*_0_, *S*_1_, *S*_2_, *S*_3_, *S*_4_ and *S*_5,_ respectively. The applied starting voltage (*V*_s_) was 60 V and this was increased to the target voltage (*V*_t_) of 120 V at a rate of 0.1 Vs^−1^ [8]. The reaction temperature was fixed at 0°C. The samples were first anodized at *V*_s_ for 10 min. Then, the voltage was raised to *V*_t_ and the samples were anodized for another 3 min. The morphology of AAOs was characterized by field-emission scanning electron microscopy (SEM) with images collected from the bottom part of the produced AAOs after wiping and subsequently etching the Al for 55 min in 5 wt% H_3_PO_4_ solution at 45°C. The fast Fourier transforms (FFTs) of the SEM images were carried out by WSxM 5.0 [16].

## 3. Results and discussion

**Figure 1.**
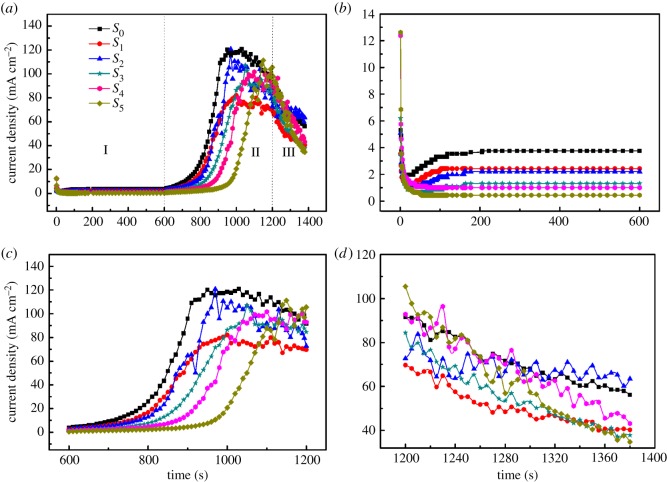
The current density versus time curves for the studied samples. (*b*–*d*) Larger magnifications of the stages in (*a*) (I, II and III, respectively).

Figure 1 presents the time-dependent current density (*j*_a_) of *S*_0–5_ samples. As shown in figure 1*a*, the changes in *j*_a_ are divided into three stages. Stage I: the *j*_a_ decays fast and reaches a minimum in a short time. Subsequently, it increases to a peak in the peak time followed by a slight decrease and then arrives at a stable value (*j*_s_). This phenomenon can be explained by Liu's current theory on the pore-growth model of AAO [17]. Stage II: firstly, the *j_a_* elevates due to the increasing voltage with a rise in the transport of ions passing through the barrier layer. Next, it decreases slowly with a large amount of fluctuation. This is because a high voltage creates a thicker barrier [16], and a slight decline in the number of ions passing through the barrier layer results in a smaller current. Stage III: The *j*_a_ decreases gradually. This is because the level of oxygen ions mostly coming from water decreases in the electrolyte according to the reaction (H_2_O(*l*) → O + 2H^+^(*aq*)* *+ 2e^−^) [14]. In the meantime, there is greater volatility, possibly because the heat exchange enhances the velocity of the ions in the local electrolyte.

Figure 1*b* reveals that the higher EG concentrations lead to lower *j*_s_. The effect can be explained by the formula [18]

$$j=\frac{Fc\zeta \varepsilon \text{E}}{4\pi \eta },$$

where *j*, *F, c, ζ, ε, Ε* and *η* represent the current of ions, the Faraday constant, ionic concentration, zeta potential, dielectric coefficient, electric field strength and the viscosity coefficient, respectively. The reduction of the *ε* and increase of the *η* of the electrolyte due to the addition of EG results in the lower *j*_s_. Meanwhile, the phenomenon that *j*_a_ rises to a peak value in the peak time disappears as EG is increased because the addition of EG blocks ion diffusion. Figure 1*c* shows that the process for *j*_a_ reaching the critical value (*j*_a_ ≈ 30 mA cm^−2^) takes more time with increase in EG concentration. The increase of the reaction voltage elevates *j*_a_. Furthermore, addition of EG lowers the value of *ε*/*η,* promoting decrease in *j*_a_ for the same applied potential (*U*). It opens up the possibility for synthesis of AAO by MA (mild anodization) over a wider voltage range in an oxalic acid electrolyte containing EG.

**Figure 2.**
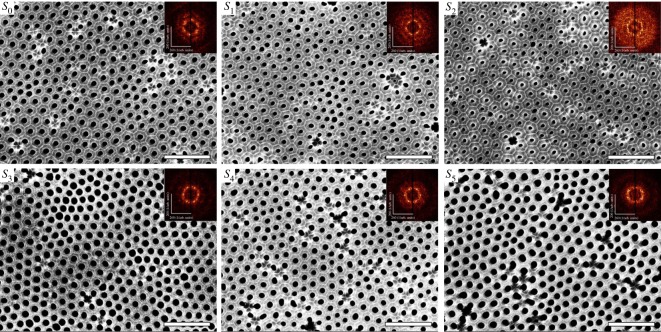
SEM images of the bottom part of the AAO samples along with the FFT images. Scale bars = 1 µm.

SEM images of the bottom part of the analysed AAO along with their FFTs images are exhibited in figure 2. The FFTs of the lattice present the periodicity of AAO in the inverse scale. An FFT pattern consists of six distinct spots for a perfect triangular lattice. Ring-shaped forms could be observed in the FFT image for the worst lattice. It is shown that EG has an obvious effect on AAO quality. The pore ordering gradually decreases with increase in EG concentration to 20 wt%. In the *S*_2_ sample, the hexagonal pore ordering almost disappeared. This is probably because the heat exchange rate lags behind the growth rate of the AAO. However, the pore ordering was improved with EG concentration exceeding 20 wt%. It is likely that the local heat exchange accelerates the dissolution of the pore wall. This reveals that it is necessary to mix EG into the aqueous electrolyte during preparation of AAO by HA, which is a completely new perspective. The pore ordering is also well determined by the FFT rings which form distinct points at the corner of a hexagon for the considerably well-ordered AAO.

**Figure 3.**
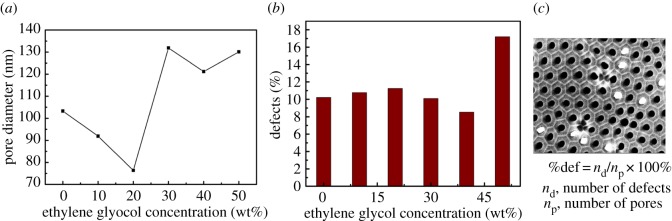
The pore diameter (*D*_p_) (*a*), the number of estimated defects (*b*) and the scheme demonstrating the determination of percentage of estimated defects (*c*) for the studied samples.

The pore diameter (*D*_p_) and the number of estimated defects per given area in accordance with stacking faults in hexagonal pore arrangement are shown in figure 3. One can observe that *D*_p_ showed a nonlinear relation with EG concentration. The *D*_p_ presents a downward trend for the EG concentration under 20 wt%. It can be explained that the EG molecules play an important role in inhibiting the chemical dissolution of alumina during anodization when the EG concentration is less than 20 wt% [19]. However, the *D*_p_ shows a sharp rise when the EG concentration exceeds 20 wt%. There could be two reasons for this. First, it is well known that pore distribution and size are determined by many factors including voltage, electrolyte concentration, reaction temperature, solution viscous flow, etc. The solution viscous flow changes after adding EG with a different mass ratio in the range of 10–50 wt% [20]. In our opinion, viscous flow has a great effect on the internal heat exchange between solution and AAO substrate. It is concluded that the addition of EG produces both the positive factors, such as decreasing permittivity of the electrolyte and inhibition of the chemical dissolution of alumina during anodization, and negative factors, such as slowing down of the heat exchange due to relatively higher solution viscous flow. The *D*_p_ rapidly increases as the EG concentration exceeds 20 wt%. This is probably because the effect of heat exchange overshadowed the role of EG that inhibits the chemical dissolution of alumina during anodization; Second, extensive incorporation of soluble ${\text{C}}_{2}{\text{O}}_{4}^{2-}$ and COO^−^ ions into the oxide framework probably favours cleavages and enhances the oxide dissolution rate along the cell boundaries [15].

In figure 3*b,c*, the number of estimated defects increases slowly for the *S*_0_, *S*_1_ and *S*_2_ samples. However, it dramatically increases as the EG concentration reaches 50 wt%. This is probably because 50 wt% EG added to electrolyte would induce drastic heat exchange.

To better characterize the order degree of AAO, semi-quantitative analysis of the regularity parameter (*R*) from the FFT's intensity profile was performed [13], which could be represented by the following formula:

$$R=\frac{H}{W_{1/2}}.$$

**Figure 4.**
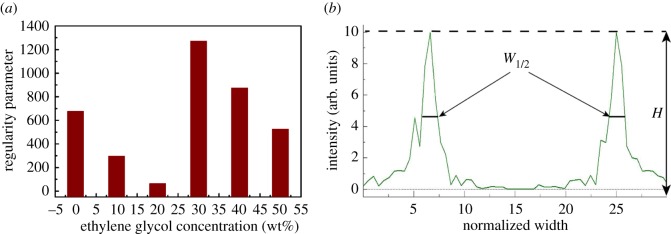
Plot of the regularity parameter (*R*) (*a*) and scheme demonstrating the intensity profile analysis of the FFT (*b*) against EG concentration for the studied samples.

Here the regularity parameter is defined as the ratio of intensity to the full width at half maximum of the FFT's intensity profile. The corresponding *R* versus EG concentration is presented in figure 4. Significant growth of *R* is observed as the EG concentration reaches 30 wt%. Perhaps this is because the heat exchange is in balance with the dissolution of the pore wall. Briefly, we can conclude that the pore ordering is related to the presence of EG.

## 4. Conclusion

In this work, the influence of EG on the pore arrangement of AAO fabricated by HA in oxalic acids was investigated. It was demonstrated that EG has a substantial impact on the pore arrangement in AAO. A long-range ordered AAO can be obtained when the sample is anodized in a 30 wt%-EG-containing oxalic acid electrolyte. Moreover, minimum *D*_p_ can be gained with an EG concentration of 20 wt%. The number of estimated defects of AAO increases sharply as the concentration of added EG reaches 50 wt%.

## Data accessibility

Our data are available from the Dryad Digital Repository: http://dx.doi.org/10.5061/dryad.60n2m [21].

## References

[1] Chu SZ, Inoue S, Wada K, Kurashima K 2005 Fabrication of integrated arrays of ultrahigh density magnetic nanowires on glass by anodization and electrodeposition. Electrochimica. Acta. 51, 820–826. (doi:10.1016/j.electacta.2005.03.075)

[2] Ghicov A, Schmuki P 2009 Self-ordering electrochemistry: a review on growth and functionality of TiO_2_ nanotubes and other self-aligned MO_x_ structures. Chem. Commun (Camb). 10, 2791–2808. (doi:10.1039/b822726h) 10.1039/b822726h 19436878

[3] Ren Y, Ma Z, Bruce PG 2012 Ordered mesoporous metal oxides: synthesis and applications. Chem. Soc. Rev. 41, 4909–4927. (doi:10.1039/c2cs35086f) 22653082 10.1039/c2cs35086f

[4] Vega V, Javier G, Montero-Moreno JM, Hernando B, Bachmann J, Prida VM, Nielsch K 2015 Unveiling the hard anodization regime of aluminum: insight into nanopores self-organization and growth mechanism. ACS Appl. Mater. Interfaces 7, 28 682–28 692. (doi:10.1021/acsami.5b10712) 10.1021/acsami.5b10712 26646814

[5] Md Jani AM, Losic D, Voelcker NH 2013 Nanoporous anodic aluminium oxide: advances in surface engineering and emerging applications. Prog. Mater. Sci. 58, 636–704. (doi:10.1016/j.pmatsci.2013.01.002)

[6] Jeong SH, Im HL, Hong S, Park H, Baek J, Park DH, Kim S, Hong YK 2007 Massive, eco-friendly, and facile fabrication of multi-functional anodic aluminum oxides: application to nanoporous templates and sensing platforms. RSC. Adv. 7, 4518–4530. (doi:10.1039/C6RA25201J)

[7] Sarkar J, Khan GG, Basumallick A 2007 Nanowires: properties, applications and synthesis via porous anodic aluminium oxide template. Bull. Mater. Sci. 30, 271–290. (doi:10.1007/s12034-007-0047-0)

[8] Lee W, Park SJ 2014 Porous anodic aluminum oxide: anodization and templated synthesis of functional nanostructures. Chem. Rev. 114, 7487–7556. (doi:10.1021/cr500002z) 24926524 10.1021/cr500002z

[9] Norek M, Dopierała M, Bojar Z 2016 The influence of pre-anodization voltage on pore arrangement in anodic alumina produced by hard anodization. Mater. Lett. 183, 5–8. (doi:10.1016/j.matlet.2016.07.038)

[10] Sachiko O, Makiko Z, Miyuki I, Hidetaka A 2004 Controlling factor of self-ordering of anodic porous alumina. J. Electrochem. Soc. 151, 473–481. (doi:10.1149/1.1767838)

[11] Schwirn K, Lee W, Hillebrand R, Steinhart M, Nielsch K, Gösele U 2008 Self-ordered anodic aluminum oxide formed by H_2_SO_4_ hard anodization. ACS. Nano. 2, 302–311. (doi:10.1021/nn7001322) 19206631 10.1021/nn7001322

[12] Zaraska L, Sulka GD, Jaskuła M 2010 The effect of n-alcohols on porous anodic alumina formed by self-organized two-step anodizing of aluminum in phosphoric acid. Surf. Coat. Technol. 204, 1729–1737. (doi:10.1016/j.surfcoat.2009.10.051)

[13] Chen W, Wu J-S, Xia X-H 2008 Porous anodic alumina with continuously manipulated pore/cell size. ACS. Nano. 2, 959–965. (doi:10.1021/nn700389j) 19206493 10.1021/nn700389j

[14] Martin J, Manzano CV, Caballero-Calero O, Martín-González M 2013 High-aspect-ratio and highly ordered 15-nm porous alumina templates. ACS. Appl. Mater. Interfaces 5, 72–79. (doi:10.1021/am3020718) 23215033 10.1021/am3020718

[15] Norek M, Stępniowski WJ, Siemiaszko D 2016 Effect of ethylene glycol on morphology of anodic alumina prepared in hard anodization. J. Electroanal. Chem. 762, 20–28. (doi:10.1016/j.jelechem.2015.12.026)

[16] Hillebrand R, Muller F, Schwirn K, Lee W, Steinhart M 2016 Quantitative analysis of the grain morphology in self-assembled hexagonal lattices. ACS. Nano. 2, 913–920. (doi:10.1021/nn700318v) 10.1021/nn700318v 19206488

[17] Zhang F, Liu X, Pan C, Zhu J 2007 Nano-porous anodic aluminium oxide membranes with 6–19 nm pore diameters formed by a low-potential anodizing process. Nanotechnology 18, 302–345. (doi:10.1088/0957-4484/18/34/345302)

[18] Lee W, Ji R, Gösele U, Nielsch K 2006 Fast fabrication of long-range ordered porous alumina membranes by hard anodization. Nat. Mater. 5, 741–747. (doi:10.1038/nmat1717) 16921361 10.1038/nmat1717

[19] Parkhutik VP, Shershulsky VI 1992 Theoretical modelling of porous oxide growth on aluminium. Appl. Phys. 25, 1258–1263. (doi:10.1088/0022-3727/25/8/017)

[20] Houser JE, Hebert KR 2009 The role of viscous flow of oxide in the growth of self-ordered porous anodic alumina films. Nat. Mater. 8, 415–420. (doi:10.1038/nmat2423) 19363477 10.1038/nmat2423

[21] Guo Y, Zhang L, Han M, Wang X, Xie J, Deng L 2018 Data from: The effect of ethylene glycol on pore arrangement of anodic aluminium oxide prepared by hard anodization Dryad Digital Repository. (http://dx.doi.org/10.5061/dryad.60n2m) 10.1098/rsos.171412 PMC5882678 29657754

